# Consumer Understanding, Perception and Interpretation of Serving Size Information on Food Labels: A Scoping Review

**DOI:** 10.3390/nu11092189

**Published:** 2019-09-11

**Authors:** Klazine Van der Horst, Tamara Bucher, Kerith Duncanson, Beatrice Murawski, David Labbe

**Affiliations:** 1 Department of Health Professions, Bern University of Applied Sciences, 3005 Bern, Switzerland; klazine.vanderhorst@bfh.ch; 2 Société des Produits Nestlé S.A., Nestlé Research, Institute of Material Science, 1000 Lausanne, Switzerland; 3 School of Health Sciences, Faculty of Health and Medicine, The University of Newcastle, Callaghan, NSW 2308, Australia; tamara.bucher@newcastle.edu.au (T.B.); kerith.duncanson@newcastle.edu.au (K.D.); 4 Priority Research Centre for Physical Activity and Nutrition, The University of Newcastle, Callaghan, NSW 2308, Australia; beatrice.murawski@newcastle.edu.au; 5 School of Medicine and Public Health, Faculty of Health and Medicine, The University of Newcastle, Callaghan, NSW 2308, Australia

**Keywords:** serving size, portion size, food labeling, nutrition facts label, back of pack, front of pack, health framing

## Abstract

The increase in packaged food and beverage portion sizes has been identified as a potential factor implicated in the rise of the prevalence of obesity. In this context, the objective of this systematic scoping review was to investigate how healthy adults perceive and interpret serving size information on food packages and how this influences product perception and consumption. Such knowledge is needed to improve food labelling understanding and guide consumers toward healthier portion size choices. A search of seven databases (2010 to April 2019) provided the records for title and abstract screening, with relevant articles assessed for eligibility in the full-text. Fourteen articles met the inclusion criteria, with relevant data extracted by one reviewer and checked for consistency by a second reviewer. Twelve studies were conducted in North America, where the government regulates serving size information. Several studies reported a poor understanding of serving size labelling. Indeed, consumers interpreted the labelled serving size as a recommended serving for dietary guidelines for healthy eating rather than a typical consumption unit, which is set by the manufacturer or regulated in some countries such as in the U.S. and Canada. Not all studies assessed consumption; however, larger labelled serving sizes resulted in larger self-selected portion sizes in three studies. However, another study performed on confectionary reported the opposite effect, with larger labelled serving sizes leading to reduced consumption. The limited number of included studies showed that labelled serving size affects portion size selection and consumption, and that any labelled serving size format changes may result in increased portion size selection, energy intake and thus contribute to the rise of the prevalence of overweight and obesity. Research to test cross-continentally labelled serving size format changes within experimental and natural settings (e.g., at home) are needed. In addition, tailored, comprehensive and serving-size-specific food literacy initiatives need to be evaluated to provide recommendations for effective serving size labelling. This is required to ensure the correct understanding of nutritional content, as well as informing food choices and consumption, for both core foods and discretionary foods.

## 1. Introduction

The food environment in which people select, prepare and consume food has changed considerably in recent years. Improvements to agricultural practices, food transportation, food processing, and food storage have contributed to an increase in food availability and variety [1]. A decrease in home-prepared foods and increased purchasing and consumption of packaged foods has led to increased reliance on food package labels including information about the composition of foods purchased and consumed [2,3,4]. In parallel, an increase in portion size for packaged food and beverages has been identified as a potential factor contributing to the rise of the prevalence of obesity between 1977 and 2006 in the United States of America (USA) [5]. The influence of the changing food environment on weight status resulted in increased investigation of this association in the literature [6]. The factors that influence food choice as a behavior in this abundant food environment are likely to be mediated by attitudes and beliefs at an individual level, as described by Ajzen’s Theory of Planned Behavior [7,8].

In this context, the importance of nutritional information labelling including serving sizes is paramount for consumer awareness and understanding of their food purchasing and in guiding them toward informed food choices and portion size selection.

The term “serving size” pertains to the labelled serving size found on a food label [9], unlike “portion size”, which describes the actual amount of food that has been consumed [10]. However, the terms “serving size” and “portion size” are often used interchangeably, which may lead consumers to believe they mean the same thing, despite this distinct difference. This misconception has led to confusion related to serving sizes on labels, which was originally intended to guide food selection and portion sizes [11,12,13].

In 35 countries (including European countries, USA, China, Brazil, Japan, Australia), a nutritional information panel on food packages is mandatory, and legislation requires or recommends the listing of nutritional information on a serving size basis [14]. Serving size information, which should represent the amount customarily consumed, is either regulated (e.g., USA, Canada) [15] or determined by the food manufacturers (e.g., in Australia and in European countries) [14]. Thus, serving sizes can vary between products in the same food category and with the same volume [16,17]. At a conceptual level, the “per serving” information is useful for consumers to estimate how much of a nutrient they are consuming. For example, if an individual with cardiovascular disease is monitoring fat consumption, they may use the “per serving” amount to help calculate their daily total fat intake from packaged foods [18].

In May 2016, the U.S. Food and Drug Administration (FDA) announced a new nutrition facts label for packaged foods to reflect new scientific information, including the link between diet and chronic diseases such as obesity and heart disease [15]. This new regulation included updates on serving sizes and labelling requirements for certain package sizes. As the portion sizes consumed have increased within the last decade [19], these regulations were updated. For packaged foods that contain up to 200% of the reference amount customarily consumed (RACC), such as a 20 ounce (600 mL) soda or a 15 ounce (425 g) can of soup, calories and other nutrients will now be required to be labelled as one serving, because a person typically consumes this amount in one sitting. These specified serving sizes tend to be similar to serving sizes in the national level food guidance systems, but are not exactly identical, which adds another layer of complexity and confusion for consumers. The confusion regarding the current standards for serving sizes used on packages as well as the advice provided to guide portion sizes (i.e., how much should be consumed) among consumers is partially due to the heterogeneity in rules and regulations surrounding serving sizes as well as inconsistencies in the terminology used.

The literature provides mixed information on consumer understanding and use of food labels. Several reviews are available that explore the consumer understanding of labelling. These reviews report that most consumers looked at nutrition labels “often” or “sometimes”, with some participants indicating that labels influence their food purchases [20]; that consumers lack understanding with regard to some nutrition label terms [20,21,22]; or that there is a potential positive effect of front-of-pack labeling in guiding consumers’ choices towards healthier products [23,24]. Low health literacy is associated with less food label use and poorer diet quality [25], as well as less accurate estimates of serving sizes [26]. The heterogeneity in presenting serving sizes, and hence nutritional information for similar foods, compromises its efficiency in guiding consumers toward informed food choices [14,16]. Additionally, a tendency has been identified whereby foods with a higher calorie density are displayed using smaller serving sizes. This further increases the complexity and limits the usefulness of nutritional information from a consumer perspective [17], while consumers also feel conflicted with inconsistent messages about what and how much they should eat [27]. The evidence shows that consumers obtain information regarding portion sizes from a number of sources including dietitians and food packages, much of which can be contradictory or inconsistent [27]. Consumers describing the burden of deciphering food labels and how this causes misinterpretations of portion size guidance also tend to perceive the serving sizes provided (e.g., cereal) as too small and not relatable to the amounts consumed [27]. One suggestion of how healthy portion size choices and consumption could be promoted concerns the manipulation of labelled serving sizes [28]. This type of manipulation is called “health framing” and capitalizes on consumers’ perceptions of serving sizes. For example, food items with smaller serving sizes and nutritional information listed consequently might be considered healthier than a larger serving size of a similar food item [29]. The influence of labelled serving size information on attitudes, beliefs and resulting food choice behaviors in the current food environment has not been described within the current, abundant food environment.

The aim of this review is to provide an overview of the recent field of investigation related to consumers’ interpretation of labelled serving size information and how this influences product perception and consumption. With complex food environments and consumer confusion surrounding serving size labels [27], this knowledge is needed to inform changes to simplify food labelling and assist consumers in choosing healthy portion sizes (e.g., through improved understanding of product nutritional information).

## 2. Material and Method

The current scoping review followed a five-stage framework [30]. These stages were used to structure and guide the processes of (1) identifying a research question; (2) identifying relevant studies; (3) selecting studies; (4) charting the data; and (5) collating, summarizing and reporting the results. With the aim of addressing the research questions of how consumers interpret the labelled serving size information and how this influences product perception and consumption, the following objectives were defined.

### 2.1. Search Strategy

Seven electronic databases were used to search for relevant papers published in English: MEDLINE, The Cochrane Library, EMBASE (Excerpta Medica Database), CINAHL (Cumulative Index to Nursing and Allied Health), Scopus, PsycInfo and Business Source Ultimate. The search comprised truncated key words used individually and in combination, including “point of sale”, “point of purchase”, “nutrition/food/health/front of pack (FOP)/back of pack (BOP)” and “label/rating/symbol/information or logo”, “menu/food” and “label”, “nutrition and guideline/panel/table/profile/summary or score”, or “nutrition fact label”, “portion size”, “serve”, “serving” or “serves” (see Supplementary Materials for the full search strategy). Publications were limited to human subjects only and, where possible, a number of terms describing various diseases were excluded. Record retrieval was limited to studies published between 2010 and April 2019. These publication dates were selected to ensure currency in relation to the food environment and to calibrate somewhat in relation to serving size labelling. For example, mandatory labelling was introduced in Australia in 2002 [31] and in Europe by 2011 [32].

### 2.2. Record Screening

Results of the search were exported to EndNote X8 (Clarivate Analytics, Philadelphia, PA, US), where duplicates were removed using the inbuilt function in Endnote, which enables the automatic identification of duplicates. In addition, the identified duplicates were checked manually prior to removal. The remaining titles and abstracts were uploaded to Covidence (Veritas Health Innovation, Melbourne, Australia; available at www.covidence.org), where members of the research team were able to undertake all screening processes. The screening of titles and abstracts was shared between three reviewers, with any studies categorized as “retrieve” or “unclear” included for full-text screening. Full-text screening was conducted by two reviewers, with a third reviewer independently assessing any conflicts.

### 2.3. Selection Criteria

To guide publication selection, a set of eligibility criteria were established that aligned with the research question defined in Stage 1 (research question identification). A publication was eligible if it provided information on how consumers perceive, understand or interpret labelled serving sizes (e.g., recommended vs. usual portion), if it provided information on how the labelled serving size on food labels influences product perception, choice or consumption, or if it provided information on whether consumers differentiate between the FOP-labelled serving size and portion guidance (which is sometimes found on BOP labels) and relate to dietary recommendations such as serve sizes.

Publications were excluded if they reported information on calorie labelling on menus or the general impact of FOP labelling on consumers (i.e., not focused on serving size). Publications were also excluded if this information was not provided in the form of an on-pack label format (e.g., printed or displayed elsewhere) or did not make reference to this, or if serving size per se was not addressed on the label. Any reports on the association between physical activities and portion size on calorie-related outcomes were beyond the scope of this review, as were publications focused on any forms of portion size education other than what was provided on the label (unless strictly relating to education on serving size labelling). Publications were excluded if there was no parameter relating to consumer behavior (i.e., perception, interpretation, food choice, intake), or if the publication was merely descriptive in nature (e.g., an overview of different types of labels on the market). Publications examining packaging waste were also deemed irrelevant for this review.

### 2.4. Data Extraction

Relevant data, including the study design (e.g., study type, sample size and setting), sample characteristics (e.g., age, gender and weight), description of labels, study outcomes (including perception, interpretation and behavior) and conclusions were extracted by one reviewer into an Excel spreadsheet. A second reviewer checked the data extracted from each publication for consistency. Conflicts on study inclusion and exclusion were discussed and resolved between all authors. Extracted data were further grouped into the following sub-sections, each of which were summarized in table format:

Publication selection: authors (year); country; study type and design; sample size; description of study arms/conditions; study setting; participant age; gender ratio; and weight status.

Description of included publications: authors (year); food type; food label type; main findings relating to perception and interpretation; main findings relating to behavior; and implications.

## 3. Results

### 3.1. Publication Selection

**Figure 1:**
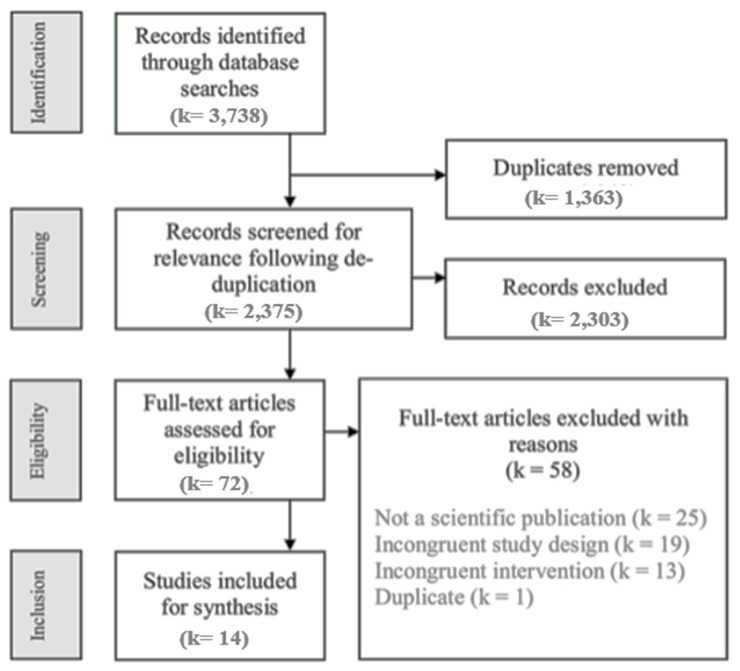
Flow diagram of study selection.

A total of 3738 publications were identified as part of the electronic database searches (MEDLINE (*k* = 644), The Cochrane Library (*k* = 36), EMBASE (*k* = 720), CINAHL (*k* = 169), Scopus (*k* = 859), PsycINFO (*k* = 222), Business Source Ultimate (*k* = 191)). Duplicates were removed (1363), which left 2375 titles and abstracts to be screened, and among them, 1793 publications were deemed irrelevant based on title and abstract screening, with disagreement resolved by a third reviewer. The remaining 72 full-text reports were assessed for inclusion by two reviewers, with conflicts resolved by discussion and consensus. Fourteen publications were included for the final synthesis (Figure 1).

The 14 papers reporting findings from 29 studies (nested experiments) were published between 2012 and 2019. Studies took place in four different countries, including ten from the USA [33,34,35,36,37,38,39,40,41], two from Canada [42,43], one from Australia [44], and one from the United Kingdom [45]. Sample sizes across these studies ranged from *n* = 51 [39] to *n* = 16,048 [40], including nine studies with less than 1000 participants and four studies reporting results from 1000+ participants. Observations were either made in the form of online surveys (*m* = 8), in university settings (*m* = 5), at laboratories (*m* = 8), or held in local community settings such as at college basketball game (*m* = 4) or completed at home via postal survey (*m* = 3). Table 1 provides a summary of descriptive data for each of the included studies.

### 3.2. Description of Included Studies

Participants: Studies recruited adult volunteers either from the general public (*k* = 8) or university students (*k* = 5), and one study used purchase transaction data from a food retailer (*k* = 1). All but one sample [44] were mixed gender. One study [40] did not report a gender ratio but examined gender as a moderator. The average participant age per sample ranged from 18.0 years [34,44,46] to 75.0 years [34]. Measures of body mass index (BMI) or weight status were provided for six of the samples, which ranged from 21.5 to 28.5 [33,36,41,42,43,44]. For the remaining eight samples, no weight status was reported. None of the studies excluded individuals from participating based on this criterion.

Study designs: Various study designs were employed to answer respective research questions, with experimental studies involving between two and 10 comparator conditions. A non-randomized experimental design was used in three studies, none of which had a control group [33,35,37]. An experimental survey design (random allocation, no control group) was used in four studies [36,38,42,46]. A randomized controlled trial (RCT) design was chosen for three studies, either using three study arms [41,43] or four study arms [44]. A cross-sectional design was used in four studies [34,39,40,45].

Test conditions, comparator conditions and measurement of consumer perception, interpretation and behaviors: All of the included studies involved consumers reporting on serving size information on food packaging via a paper-based [34,37,43] or online survey [33,36,38,42,46] with the use of food models described in five of the papers [33,34,35,39,46]. Eight experiments/surveys specifically provided BOP nutrition facts and serving size labelling [33,34,36,37,40,42,43,44], and three provided both FOP and BOP nutrition facts and serving size labelling [35,38,39]. Seven papers reported having selected discretionary foods to be studied [34,35,36,37,41,42,44], five used both discretionary and core foods [33,38,39,45,46], and two studies involved the use of generic food labels [40,43].

Consumer perception and interpretation (including understanding, beliefs and concerns) about nutrition facts and serving sizes on existing labels were investigated in three studies [34,36,38] with a focus on the influence of health framing on consumer perception; i.e., how serving size affects nutritional information and related anticipated guilt after eating the product [38]. Seven studies investigated consumer understanding of proposed or modified nutrition facts labelling and serving size information in comparison to existing ones [33,35,37,39,40,41,42]. How consumers interpreted nutrition facts according to the number of servings per pack and the size of the pack was considered in one study [43].

Five articles investigated consumer behaviors in relation to proposed or modified nutrition facts labelling and serving sizes [33,35,39,41,44]. The influence of health framing on purchasing intention was also investigated [38], as were purchasing behaviors before and after the introduction of recommended serving sizes on nutrition labels [45], and the impact of varying granularity (i.e., fine-grained vs. gross-grained labels) of serving size information on intended and actual consumption and portion size perception [46].

### 3.3. Description of Study Findings

The 14 publications selected for inclusion in this scoping review related to a range of research questions and hypotheses. However, the studies were sufficiently consistent in design and measures to be consolidated into a set of study findings, as they were concerned with either the perception and interpretation, or behaviors (purchase, consumption) in relation to the labelled serving size. Table 2 summarizes the findings by study.

Consumer health perception of labelled serving size: Consumer health perceptions towards serving size labelling were measured in different ways in the studies that reported on this influence. In one study, serving size decreased product-related health perception (*p* < 0.001) and increased guilt associated with consumption (*p* < 0.05) but was perceived as more representative of portions typically consumed (*p* < 0.05 all foods) [35].

Two studies reported a negative impact in relation to consumer perception of serving size labelling. In a study specifically related to the health framing of labelling, the manipulation of serving size (and nutritional) information through health framing (i.e., reducing serving size) reduced consumption guilt (*p* < 0.05) for consumers who were more concerned about their diet [38]. These findings were consistent with the study that used a real-world setting in which a reduction of the labelled recommended serving size by 50% increased sales volume by an average of 4% in the yogurt category, with an even more pronounced effect when the serving-size specification was particularly small [45]. In the open response section of a large national cross-sectional survey reported, a small subsample of participants expressed distrust of serving size information [40].

Consumer understanding and interpretation of labelled serving size: Improved accuracy in serving size estimations is associated with higher numeracy, nutrition knowledge, and self-reported food label use and is enhanced by the provision of detailed instructions, even for difficult comparisons in which per serving and per package information was inconsistent [37]. Conversely, serving size estimation in this study was compromised by poorer numeracy (all ages), poor attention skills, and fewer instructions (older adults only).

Three studies investigated consumer interpretation of labelled serving size and identified that consumers interpret serving size as a recommended serving rather than as a typical serving [33,37,40]. A discrepancy between the understanding of serving size and portion size was reported, with 78% of participants believing that serving size related to how much food can or should be consumed in one sitting as part of a healthy diet [33]. In a cross-sectional study (*n* = 16,280) the majority of respondents misinterpreted the meaning of serving size, particularly women and obese individuals [40]. Indeed, about half the respondents reported that serving size is “the amount of this food that people should eat’’ rather than an amount that ‘‘people usually eat’’ or ‘‘that makes it easier to compare foods.’’ In a recent experimental study, it was shown that reported accuracy in serving size interpretation was also low (50–55%) across all age groups [37].

In two studies that compared existing to modified versions of serving size labelling, accuracy in calorie estimation was improved with a nutrition label that contained both serving size per serving and per-container (dual column information) [42]. Dual column information has also been shown to improve accuracy for complex calorie estimation tasks [36]. Participants of another study had difficulties in estimating total nutrients and calorie content present in a four-serve ice-cream container based on nutrition facts provided for one serving. The authors recommended dual column nutritional information to improve understanding of nutritional information [34]. In the same study, participants with higher scores on nutritional information understanding consumed less soda. While there was no association between different serving size display formats (e.g., font size or order) and correct energy estimation, the majority (62%) of participants preferred a serving size format that included servings per package [42]. In a study that investigated food image depiction on the front of packages, the authors identified that portion size depictions (i.e., the image of the cereal bowl on cereal boxes) were 64.7% larger than the portions recommended on the nutrition facts label [39].

When a product was presented as a single serving pack, but actually contained multiple servings, participants made significantly more serving size assumption errors compared to when a pack was not misleading (i.e., a single pack containing a single serving and a multi serving pack containing multiple servings [43]).

Consumer behavior in relation to labelled serving size: The behaviors specific to labelled serving sizes exhibited by participants in the included studies were influenced by a range of factors, including understanding of food labelling, health framing, and intentional modification to labelling. Three articles reported increased portion sizes as a result of using larger serving sizes [33,39,44]. Modified (larger amount) serving sizes on labels relative to existing serving sizes led consumers to serve themselves 41% more cookies, serve 27% more cheese crackers to another person, buy 43% more lasagne for others and divide a lasagne into 22% larger slices [33]. Similarly, cereal boxes that depicted exaggerated serving sizes (i.e., a cereal bowl with a large portion on the package illustration) resulted in 17.8% more cereal being portioned compared to boxes that depicted a single-size portion and 42% more than the suggested serving size [39]. Labelling pizza with a higher number of servings decreased food intake relative to labelling the pizza with a lower number of servings (*p* < 0.05) [44]. In contrast, consumers who viewed larger serving sizes ate less confectionery than those presented with the current serving sizes (*p* < 0.05), and larger serving sizes led to an overestimation of calories and greater anticipated guilt (*p* < 0.05) [35].

Health framing influenced behaviors as well as perception and serving size interpretation [38]. Health framing seemed to reduce the anticipated guilt associated with consuming calories, enabling consumers who were concerned about their diet to form stronger purchase intentions (*p* < 0.05). FOP labels assisted consumers to better estimate calories per serving, but this improved knowledge did not influence perceptions of healthfulness, taste, purchase intent, or the amount of cereal poured or consumed [41]. A notable finding was a trend towards a significant positive effect of unhealthier purchases in terms of calories per 100 g after label introduction, indicating that consumers react differently to the health framing of nutritional information depending on the “healthiness” of products [45].

High granularity (e.g., 15 pieces of chips) in describing serving sizes relative to low granularity (e.g., one serving of chips) decreased both the intended and the actual intake of the labelled food [46]. High granularity serving size description increased the perceived food size (i.e., people considered the food as larger, weighing more, costing more, and containing more calories), which reduced intake. Low granularity serving size description showed the reverse.

Definitions of serving size: Different interpretations of serving and portion sizes were used across the studies. For example, Dallas, Liu and Ubel [33] reported that “the correct definition of serving size is the amount that people typically consume in one sitting” and an “incorrect definition of serving size is the amount of the product that can or should be consumed in one sitting as part of a healthy diet” [33]. This study was included as it was apparent that the influence of the labelled serving size was examined, although the working definition used in this study was unfitting. A further example of differing terminology was evident in a study demonstrating that “portion size depictions on FOP of breakfast cereal boxes are 64.7% larger than recommended portions on the nutrition facts label” [39]. The terminology used in two studies [33,39] differed from each other and from all other included studies, in that serving size referred to the manufacturer-set amount listed in conjunction with nutrition facts on labels, and portion size was the commonly consumed amount. It should also be noted that the study on breakfast cereal [39] referred to portion size images in terms of photographs of a cereal bowl, which is part of packaging design rather than a FOP label.

## 4. Discussion

This scoping review was undertaken to identify how consumers interpret labelled serving size information and how this influences product perception and consumption. The study aim was to provide recommendations for effective serving size display to ensure the correct understanding of product nutrition information and inform product choices, leading to a healthier diet.

The results of this scoping review highlight some key points for consideration in relation to the serving size labelling of food products and their relationship to usual consumption (portion size). Consumers tended to interpret the labelled serving size as a recommended serving size rather than a typical portion size [33,37,40] and to inaccurately estimate nutritional content per serving [34,36,42]. The incorrect or inaccurate interpretation of serving size was exacerbated by demographic characteristics (age, sex, education level) and weight status [37,40]. Findings showed that serving size estimation accuracy was enhanced by the provision of detailed instructions, even for difficult and inconsistent servings and per package information. This provides an indication that improvements to consumer food label literacy are an important focus for serving size labelling [37]. Overall, consumers interpreted recommended serving size information as indicative of nutrient consumption without following recommendations to inform portion size [41]. The theoretical interpretation of the findings of this review are highly consistent with Ajzen’s Theory of Planned Behavior [7]. It is evident that the beliefs of the individual regarding recommended serving size information influenced their behavior, resulting in a larger portion being served. Labelling a product with both serving size and dual column information (per serving and for the whole pack) was preferred by consumers [42] and avoided confusion to extrapolate nutrition facts for one serving to the entire content of a multi serve pack product [34]. A dual column format is commonly used and widely accepted in food labelling [47] and has previously been reported to improve understanding by providing a contextual cue [48]. For this combination of labelling to be relevant and useful to consumers, appropriate serving size information against which to benchmark nutrient levels is necessary.

In general, the perceptions of consumers could be influenced by the manipulation or framing of serving size information, with evidence of demographic influences on susceptibility to misleading serving size information. Larger serving sizes were generally perceived as more realistic portions than smaller serving sizes, as these were perceived as unrealistic. This finding provides support for the changes to legislation such as those that have been implemented in North America [15] from the perspective of consumer approval and support. However, this may encourage consumers to eat more if serving size is understood as the recommended portion.

The impact of serving size information on consumer portion size varied between studies and between study foods and whether these were considered discretionary or core foods. These findings suggest that different reference information or conditions may need to be applied to core and discretionary foods. Further investigation is also needed to explore the influence of the health framing that results from the application of serving size information to other parts of BOP and FOP labelling; of particular importance is improving the understanding of the impact of health framing on “healthier” compared to “unhealthier” foods, especially in relation to food purchasing behaviors [45]. Moreover, alternative portion guidance labels could have a potential health framing and consumption effect, as was found in the five-a-day portion guidance label for fruit and vegetables. A study revealed significantly lower subsequent fruit and vegetables consumption using smoothies displaying the “3 of your 5-a-day” label compared to the “1 of your 5-a-day” label. This highlights the importance of examining actual product consumption and also indicates that the daily intake of certain food groups might be influenced by labelling [49]. From a theoretical perspective, the influence of health framing on perceptions about the healthfulness of foods aligns with the attitudes component of the Theory of Planned Behavior. Food choice behavior is mediated by the attitude of the consumer, which has been influenced by how the product has been framed [8].

While FOP labelling was considered helpful to consumers, it performed better for tasks that related to product choice based on perceived healthfulness rather than serving size estimation [37]. FOP serving size labelling could therefore be considered to be relevant for product selection; for instance, using a pictorial serving size recommendation instead of an amount in grams to more efficiently inform consumers with poor numerical literacy [50]. Providing more granularity in serving size information on FOP labels for unhealthy and countable food items could also have a positive influence on consumption, whereas less granularity in serving size information could promote the consumption of healthier foods [46]. BOP serving size information can subsequently be used to inform customers about how much to purchase and consume based on dual column information.

Further research in ecological environments (e.g., at point of sale, in the home) is required to provide recommendations for effective serving size labelling to ensure the correct understanding of nutritional content and informed food choice and consumption. It is important for future research to investigate the impact of the labelled serving size on consumption of specific core foods and on discretionary foods. There is a need to determine whether improved consumer serving size literacy can help overcome health framing effects for discretionary foods (e.g., a smaller serving size can increase perceived healthfulness and lead to increased intake, due to a lower calorie content per serving displayed on the pack) or if other measures are required to offset the influence of health framing, particularly for susceptible consumer groups. Promising strategies to increase serving size literacy reported in the scoping review include comparative information on nutrition facts labels, realistic serving sizes and a comparison to standard reference amounts; for example, from national food guidance systems or the use of international food volume units [51].

## 5. Limitations

The results of this scoping review need to be evaluated while taking into account several limitations. As 12 out of the 14 papers were conducted in North America, the results need to be contextualized to consider the change in serving size labelling legislation [15] in North America in May 2016, as most studies were conducted in the preceding four years or immediately after this time-point. These changes were intended to ensure that consumers were aware of the nutritional composition of foods they were consuming, using a more standardized and realistic food amount than previously indicated on serving size labels. Therefore, cross-cultural research is required including countries where serving size labelling is not regulated.

The majority of included studies for which weight status was measured predominantly involved participants with a healthy weight status. This is important as overweight and obesity have the potential to influence serving size perception, interpretation and behaviors, and thus, the weight status of study populations needs to be accounted for [52]. Studies were mainly conducted in lab environments, and it would be useful for future research on influences of serving size labelling on food choice and consumption to be conducted in more ecologically valid settings such as at the point of sale or in the home. This is increasingly feasible in the current research environment with the increasing availability of technologies such as wearable cameras that can monitor behaviors [53]. Therefore, the results of the scoping review are synthesized in light of the rapidly changing food labelling landscape, different serving size legislation between countries, changes to labelling legislation in some countries during the selected search period (2010–2019), and the possible implications of increasing or standardizing serving sizes and the environments in which studies were conducted.

The terms “serving size” and “portion size” appear to be used inconsistently in the scientific literature. The present review may have excluded a number of findings from research that used the term “portion size” but in fact examined how different “serving sizes” influence consumer perception or behaviors. However, it was not possible to identify such reports with sufficient consistency.

## Figures and Tables

**Table 1:** Food label serving size information scoping review: summary of included studies.

Publication	Study Design & Sample	Study/Expt.	Setting	Study Conditions/Objective	Age (Years)		Gender (% m/f) a	BMI (kg/ht^2^)	
					M	SD		M	SD
Baxter et al. (2018) [43]	Three-arm experimental design with random allocation (60 Canadian University students)	1	Laboratory (*n* = 20)	Consumer interpretation of nutrition facts table using single serving (i.e., smaller) pack size containing multi serving (SSMS)	20	3.0	55/45	24.7	3.9
		2	Laboratory (*n* = 20)	Consumer interpretation of nutrition facts table using single serving (i.e., smaller) pack size containing one serving (SSSS)	20	2.0	41/60	24.9	4.9
		3	Laboratory (*n* = 20)	Consumer interpretation of nutrition facts table using multi serving (i.e., larger) pack size containing multi serving (MSMS)	19	6.0	53.8/45.2	23.6	3.5
Dallas et al. (2015) [33]	Nested experimental design (273 U.S. adults)	1	Online (*n* = 101)	Consumer interpretation of the meaning of SS information	32.5	10.8	55.3/44.7	26.2	5.78
		2	College Basketball game (*n* = 51)	Influence of exposure to current vs. proposed SS on food portions participants serve themselves	34.0	11.3	58.8/41.2	25.4	4.74
		3	University marketing course (*n* = 60)	Influence of exposure to current SS labelling on food portions, served and purchased for others	20.0	1.4	53.3/46.7	21.7	3.45
		4	University marketing course (*n* = 61)	Influence of exposure to proposed SS labelling on food portions, served and purchased for others	19.7	1.5	51.8/48.2	22.0	3.39
Elshiewy et al. (2016) [45]	Cross-sectional analysis using purchase transaction data (*n* = 20 million transactions)		N/A	N/A	N/A		N/A	N/A	
Hydock et al. (2016) [35]	Nested experimental design (753 U.S. University students)	1	Laboratory (*n* = 208)	Current vs. proposed (double) SS on five different food packages in relation to perceived healthfulness and accuracy of SS depicted	32	12	54/46	N/A	
		2	Laboratory (*n* = 347)	Virtual portioning (for self) of six foods vs. label viewing to estimate own consumption, perceived healthfulness, calorie content and consumption guilt	31	10	54/46	N/A	
		3	Laboratory (*n* = 198)	Nutrition label showing current or larger SS vs. confectionery portion to assess the impact on consumption	20	1	53/47	N/A	
Jones et al. (2015) [42]	Nested experimental design with random group allocation (2011 Canadian adults)	1	Online	Beverage energy content estimation vs. per serving/per container/dual-column to test if participants correctly identify energy content	Range 16–24		50/50	22% were overweight or obese	
		2	Online	Cracker energy content vs. single serving small font/single serving large font/number of servings per bag to test if participants correctly identify energy content	Range 16–24		50/50	22% were overweight or obese	
Lando et al. (2012) [36]	Ten-arm experimental design with random group allocation (9493 U.S. Adults)		Online	Serving format: Two servings per container as single column vs. two servings per container as dual column vs. one serving per container as single-column Label format: Current Nutrition Facts label (control) vs. current label, without “calories from fat” vs. current label, without “calories from fat” and larger font vs. changed wording to emphasize there were two servings per container and “removed calories from fat” vs. dual listing for calories, with calories per serving and per container given, but remaining nutrients given only per serving and “calories from fat” removed Label content: Provision of all nutritional information per serving and per container in separate columns vs. same dual column, without “calories from fat” vs. dual column with only the calories and % DVs per serving and per container in separate columns (without “calories from fat”).Further, there were two label formats in the one serving, single-column grouping, both using a single, large serving either like the control label, but without “calories from fat” vs. one like the control label, but without “calories from fat” and larger font	46	15.5	51/49	28.5	7.1
Lewis et al., 2018 [46]	Two-arm experimental design with random group allocation (1221 US adults)	1	Public area (*n* = 80)	Impact of portion size information (1 serving vs. 11 pieces) on tortilla chips consumption intention	20.54/5.10		50/50	N/A	
		2a	Public area (*n* = 79)	Impact of portion size information (1 serving vs. 15 pieces) on gummies consumption intention and consumption	21.37/5.21		46.8/33.2	N/A	
		2b	Public area (*n* = 79)	Impact of portion size information (1 serving vs. 9 pieces) on mini rice cakes consumption intention and consumption	21.27/3.34		50.6/49.4	N/A	
		3	Online (*n* = 200)	Impact of portion size information (1 serving vs. 16 pieces) on gummies consumption intention and perceived food size	32.4/9.03		52.5/47.5	NR	
		4	Online (*n* = 160)	Impact of portion size information (1 serving vs. 16 pieces) on gummies and baby carrots consumption intention and self-regulation (with dieters)	32.23/10.84		52/48	NR	
		5	Online (*n* = 300)	Impact of portion size information (1 serving vs. 16 pieces) on self-regulation facilitation (with dieters) with a measure of regulatory struggle	34.13/11.66		54.7/55.3	NR	
		6	Laboratory (*n* = 323)	Impact of portion size information (1 serving vs. x pieces) on consumption intention, perceived food size and actual intake of carrots, gummies, potato chips, plain M&Ms, roasted and salted almonds, and seedless green grapes	34.62/16.66		31.3/68.7	N/A	
Miller et al. (2017) [37]	Pre-post experimental design (358 U.S. Community members)		Postal survey	Product pair comparison (8 items) for healthfulness, with pairs differing in SS vs. product pairs with consistent serving size to test the accuracy of serving size estimations in the context of product healthfulness	Range 20–78		40/60	N/A	
Mohr et al. (2012) [38]	Experimental between-subjects design with random allocation (151 U.S. Adults)	3b	Online	Comparison of provision of health frame (smaller SS) vs. no frame (larger SS) to examine product choice Comparison of discretionary weight (low/high) vs. product category (pizza vs. soup) with measured moderator (dietary concern, guilt) to examine product choice	46	N/A	N/A	N/A	
Persoskie et al. (2017) [34]	Repeat cross-sectional design (3165 US adults)		Postal survey	Consumer understanding of nutritional information labelling for ice-cream	N/A	N/A	48.3/51.7	N/A	N/A
Roberto et al. (2012) [41]	Three-arm RCT (216 U.S. University students)		University classroom	Original smart choices label (servings per package) vs. modified label (incl. SS) vs. no calorie label	26	10	37/63	23.2	4.5
Spanos et al. (2015) [44]	Four-arm pilot RCT (100 Australian University students)		Laboratory-based	Portion size: 200 g Pizza in 12 pieces or 400 g Pizza in 24 pieces (equal grams) Label formats: 3 × 200 g pizza (either stating “Contains 2 servings” or “Contains 4 servings” or no serving size given) and 1x 400 g pizza (no serving size given)	21	2.3	0/100	21.5	2.95
Tal et al. (2017) [39]	Observational study (51 U.S. University students)	1	University course	Comparison of FOP image with actual reported SS of 158 common cereals	N/A		N/A	N/A	
	Experimental study (51 U.S. University students)	2	University course	Comparison of varied SS (exaggerated, multiple SS vs. recommended single-SS) for two cereals in relation to pouring cereal.	22.3	N/A	31/69	N/A	
Zhang et al. (2014) [40]	Repeat cross-sectional design (16,048 U.S. adults)		Community-based surveys	Consumer understanding and use of SS information on nutrition facts in three large national surveys.	N/A		N/A	N/A	

Note. M = Mass; SD = Standard deviation; BMI = Body mass index (kilograms/height in metres ^2^); BOP = Back of pack; FOP = Front of pack; NR = Not reported; RCT = Randomized controlled trial; SS = Serving size; SSMS = Single serving pack size containing multi serving; SSSS = Single serving pack size containing one serving; MSMS = Multi serving pack size containing multi serving; a: % ratio of males/females; b: Studies 1 and 2 of this publication were deemed irrelevant for synthesis.

**Table 2:** Food label serving size information scoping review: summary of findings and implications.

Publication	Study/Expt.	Food Types	Label Types	Perception and Interpretation	Behaviour	Implications
Baxter et al. (2018) [43]		N/A	Nutrition facts table, incl. SS	Understanding nutrition facts per serving was improved for one serving per pack that appeared as a single serving (SSSS) or for a multiple serve in a multiple serve pack (MSMS) compared to a counter-intuitive small pack with multiple servings (SSMS).	N/A	“Multi serving packs lead to mathematical challenges to determine nutritional information if it seems to be a single serve”. “Small package size of multiple serve packs led participants to interpret these products as single servings, underestimating nutrient and caloric content”
Dallas et al. (2015) [33]	1	Chicken vegetable Soup	BOP nutrition facts, incl. SS	78% believed SS related to how much food can or should be consumed in one sitting as part of a healthy diet, but the proportion of participants identifying correct meaning of serving size, incorrect meaning and “other” did not differ by condition	N/A	“Increased serving sizes may lead people who use this information as a reference to serve more food to themselves and others.”
	2	Chocolate chip cookies	BOP nutrition facts, incl. SS	N/A	Modified (larger amount) label vs. current led consumers to serve themselves 41% more cookies	N/A
	3	Crackers	BOP nutrition facts, incl. SS	N/A	Modified (larger amount) label (vs. current) led consumers to serve 27% more cheese crackers to another person	N/A
	4	Lasagne	BOP nutrition facts, incl. SS	N/A	Modified (larger amount) label (vs. current) led consumers to buy 43% more lasagne for others and divide a lasagne into 22% larger slices	N/A
Elshiewy et al. (2016) [45]		Yoghurt (healthful) and cookies (unhealthful)	Guideline Daily Amount (FOP), incl. SS	N/A	Reduced SS specification increases sales volumes after label introduction in healthier category (yoghurt), but not in the unhealthy category (cookies). For example, a reduction in SS by 50% will increase sales volume by an average of 4% (yoghurt only)	“Consumers may overlook and misinterpret nutrition label information, which can result in increased consumption (health halo). Therefore, the use of FOP labels fails to promote healthy purchase behaviour.”
Hydock et al. (2016) [35]	1	Pizza; pasta; fruit loops; sliced cheese; ham	FOP and BOP nutrition facts, incl. SS	Larger SS rated lower for health perceptions *, but more representative of serving size depicted *	N/A	“Providing consumers with easier to comprehend and more accurate information on all foods served in all contexts could reduce overeating. Decreasing caloric intake, through changing perceptions of health or increasing guilt, could improve public health. Updating serving sizes on nutrition labels could help promote better dietary choices and help curb the obesity epidemic in the United States.”
	2	Macaroni cheese; chili; lasagne; rice snacks; soup; frozen fish		Larger serving sizes led consumers to perceive foods as less healthy * and estimate that their portion contained 18% more calories * and anticipate more guilt *	N/A	
	3	Confectionery		N/A	Consumers who viewed larger SS (proposed) ate less confectionery than those presented with the current SS *	
Jones et al. (2015) [42]	1	Chocolate milk	BOP nutrition facts, incl. SS	Nutrition label with per container or dual column is better for correctly identifying energy content than per serving **	N/A	“Per container and dual column increased understanding of energy content compared to per serving. This may help decrease individual consumption of DF by influencing perceptions of food health. Font size and display order of same information did not influence correct energy estimation.”
	2	Crackers	N/A	No association between SS display format and correct energy estimation. 62% preferred SS size format including servings per package	N/A	
Lando et al. (2012) [36]		Frozen meal; crisps	BOP nutrition facts, incl. SS	Single-serving per contained and dual-column formats performed better and scored higher on most outcome measures	N/A	“For products that contain 2 servings, but are usually consumed in single eating occasion, a single-serving or dual-column labelling approach is recommended.”
Lewis et al., (2018) [46]	1	Tortilla chips	1 serving vs. 11 pieces	Fine-grained label (11 pieces) decreased consumption intention vs. gross-grained labels (1 serving)		“Fine-grained label leads participants to decrease their consumption intentions and actual intake because portions are perceived to be bigger than portions described as with the gross-grained label” “Finally, granularity facilitates self-regulation of consumption,” “Highlighting for consumers the concrete number they should consume could decrease consumption of those unhealthy foods. On the other hand, it may be fruitful to do the opposite for healthy foods that people struggle to begin eating.”
	2 part a	Gummies	1 serving vs. 15 pieces	Fine-grained label decreased consumption intention vs. gross-grained labels	Fine-grained label decreased food consumption vs. gross-grained labels	
	2 part b	Mini rice cakes	1 serving vs. 9 pieces	Fine-grained label decreased consumption intention vs. gross-grained labels	Fine-grained label decreased food consumption vs. gross-grained labels	
	3	Gummies	1 serving vs. 16 pieces	Fine-grained label decreased consumption intention and increased perceived food size vs. gross-grained labels	N/A	
	4	Gummies and baby carrots	1 serving vs. 16 pieces	Fine-grained label reduced consumption intention vs. gross-grained labels for both foods Self-regulation is facilitated by fine-grained label vs, gross-grained label for gummies (unhealthy) whereas for baby carrots (healthy), label did not impact self-regulation	N/A	
	5	Candies	1 serving vs. 16 pieces	Fine-grained label reduced consumption intention vs. gross-grained labels Level of difficulty in dieting influenced consumption intention in the gross-grained condition only whereas the reducing impact of fine-grained on consumption intention was present at all levels of difficulty in dieting.		
	6	Carrots, gummies, potato chips, plain M&Ms, roasted and salted almonds, and seedless green grapes	1 serving vs. x pieces (number of pieces differed between foods)	Fine-grained label vs. gross-grained labels reduced consumption intention and perceived food size for all foods	Fine-grained label vs. gross-grained labels reduced intake for all foods	
Miller et al. (2017) [37]		Frozen pizza; snacks	BOP nutrition facts, incl. SS	Overall accuracy (i.e., ability to identify the healthiest product) was low (50–55%) across all age groups Numeracy, nutrition knowledge and self-reported food label use supported accuracy, but did not influence age differences in accuracy. Detailed instructions improve accuracy, even for difficult comparisons in which per serving and per package information is inconsistent Accuracy is compromised by poorer numeracy (all ages) and poor attention skills and with less instructions (older adults)	N/A	“Accuracy limited by lack of consideration for multiple servings rather than too many columns to evaluate or numeracy skills.”
Mohr et al. (2012) [38]		Frozen pizza; vegetable soup	FOP and BOP nutrition facts, incl. SS	Health framing manipulation reduced guilt about consumption * for consumers who were more concerned about their diet People with high dietary concern are influenced more by health framing	Health frame dietary concern affects purchase intention * and guilt mediated the influence of health framing on purchase intention for participants with high concern *	“Prevention-focused health communication influenced participants towards selection of health-framed product whereas prompting to consider calories consumed influenced choice specifically towards listed calorie count. Health communication that encouraged participants to be diligent about their diet, but wary of health framing resulted in adjustment for serving sizes and selection of product with lowest negative nutrients.”
Persoskie et al. (2017) [34]		Bulk ice-cream in container	Nutrition Facts Panel for one serving	Understanding nutrition fact information was poor, i.e., deriving calorie content in one serving for the entire container. Participants with healthier dietary habits performed better.		“To help consumers better understand serving size, dual column labels (nutritional information per serving and for the entire pack) can help”. “Schools also have a role to play in teaching students the skills they need to understand the labels and make informed dietary decisions.”
Roberto et al. (2012) [41]		Rainbow treasures cereal	FOP Smart Choices label, incl. SS	N/A	There were no significant differences between label conditions on the total amount of cereal and milk consumed	N/A
Spanos et al. (2015) [44]		Cheese pizza	BOP, incl. SS	N/A	Labelling pizza with a higher number of servings decreased food intake relative to labelling the pizza with a lower number of servings *	“Providing SS labelling on a food product can reduce the portion-size effect on consumer food intake.”
Tal et al. (2017) [39]	1	Breakfast cereals	FOP food image (photo) and BOP nutrition facts, incl. SS	Portion size depictions on front of cereal boxes 64.7% larger than recommended portions on NFL	N/A	“Biases in SS depicted on cereal packaging are prevalent and may lead to over-serving, which may consequently lead to overeating.”
	2	Breakfast cereals	FOP food image (photo) and BOP nutrition facts, incl. SS	N/A	Boxes that depicted exaggerated SS resulted in 17.8% more cereal portioned compared to boxes that depicted a single-size portion of cereal matching suggested SS and 42% more than suggested SS	
Zhang et al. (2014) [40]		Generic	BOP, incl. SS	Majority of respondents misinterpreted the meaning of SS (Surveys 2 and 3). Women and obese individuals more likely to misinterpret SS meaning. A small subsample of participants expressed distrust of SS information	Use of SS information (often or sometimes) increased from 54% to 64% from 1994 to 2008 (Survey 1). Women and obese individuals more likely to use SS often or sometimes	“The increasing use, widespread misunderstanding and distrust of SS indicates need for change to both NFL education and information.”

Note. Expt. = Experiment; BOP = Back of pack; FGS = Food guidance system; FOP = Front of pack; NFL = Nutrition facts label; OR = Odds ratio; SS = Serving size; SSMS = single serving pack size containing multi serving; SSSS = single serving pack size containing one serving; MSMS = multi serving pack size containing multi serving; N/A = Not applicable or data not available; * Mean values differed significantly from those of the comparator/control condition (*p* < 0.05); ** *p* < 0.01.

## Supplementary Materials

The following are available online at https://www.mdpi.com/2072-6643/11/9/2189/s1: detailed overview of search strings per database.
