# Supplementary material for: Consumer Understanding, Perception and Interpretation of Serving Size Information on Food Labels: A Scoping Review
Source: Nutrients. 2019 Sep 11;11(9):2189. doi: 10.3390/nu11092189 (PMC6770558; doi:10.3390/nu11092189)
Supplement: Supplementary file 1 [file nutrients-11-02189-s001.pdf]

Supplemental material

Detailed overview of search strings per database

Medline

#	Searches
1	("point of sale" or "point of purchase").mp.
2	((nutrition* or food* or "front of pack*" or health* or "back of pack*") adj2 (label* or rating* or symbol* or information* or logo*)).mp.
3	((menu* or food*) adj3 label*).mp.
4	((Nutriti* adj (guideline* or panel* or table* or profil* or summar* or scor*)) or nutrition* fact* label*).mp.
5	(portion size* or serve or serving or serves).mp.
6	1 or 2 or 3 or 4
7	5 and 6
8	(animals not (humans and animals)).sh.
9	Autistic Disorder/ or autism.mp. or exp HIV/ or smoking.mp. or exp neoplasms/ or exp viruses/ or genetics/ or exp mental health/ or bioactive.mp. or osteoporosis/ or chronic pain/ or sexual.mp. or reproductive health/ or enzymes.mp. or malaria.mp. or bacterial infections/ or pathology/ or pathologic.mp. or vaccin*.mp. or immunization.mp. or zebrafish*.mp. or zebra fish.mp. or dengue.mp. or urinary.mp. or greenhouse.mp. or spores/ or kidney/ or kidney diseases/ or orthop?edic*.mp. or melanoma*.mp. or depression/ or tobacco.mp. or schizophrenia.mp. or pharmacy/ or radiology.mp. or rehabilitation/ or bacillus.mp. or chlamydia.mp. or thyroid gland/ or thyroid neoplasms/ or biobank.mp. or tooth diseases/ or genetic test*.mp. or botanical.mp. or infant mortal*.mp. or neonatal death*.mp. or neonatal mortalit*.mp. or infant death*.mp. or biometry/ or biometric identification/ or carcinoma.mp. or mercury/ or mercury.mp. or brain injur*.mp. or brain injuries/ or chloroplast*.mp. or autopsy.mp. or acquired immunodeficiency syndrome/ or pharmacological.mp. or biomedical.mp. or infectious disease*.mp. or botox.mp. or amino acid*.mp. or newborn screen*.mp. or pap test*.mp. or spectroscopy.mp. or glucometer*.mp. or yoghurt fermentation.mp. or staphylococcus.mp. or pharmacist*.mp. or domestic abuse*.mp. or catheterization.mp. or genetically modified.mp. or hypersensitivity/ or allergic reaction*.mp. or copper exposure.mp. or epilepsy.mp. or epileptic*.mp. or renal.mp. or toxicology.mp. or oral health.mp. or reproduction.mp. or chromatographic.mp. or hepatitis.mp. or fortification.mp. or endosomal.mp. or anti-inflammatory.mp. or malignant.mp. or scoliosis.mp. or transcriptome.mp. or testicular.mp. or oncology.mp. or ophthalmologist*.mp. or adhd.mp. or attention deficit disorder with hyperactivity/ or transgender.mp. or post traumatic stress*.mp. or posttraumatic stress*.mp. or infection control.mp. or pharmacometalb*.mp. or radiomics.mp. or hormonal.mp. or performance enhancing drug* performance enhancing substances/ or skin cancer*.mp. or cervical cancer*.mp. or foot ulcer*.mp. or neurocognitive.mp. or myocardial infarction.mp. or colorectal cancer*.mp. or bisexual.mp. or biomarker*.mp. or an?esthesia.mp. or ebola.mp. or anxiety.mp.
10	7 not (8 or 9)
**11**	**limit 10 to yr="2010 -Current"**

EMBASE

#	Searches
1	("point of sale" or "point of purchase").mp.
2	((nutrition* or food* or "front of pack*" or health* or "back of pack*") adj2 (label* or rating* or symbol* or information* or logo*)).mp.
3	((menu* or food*) adj3 label*).mp.
4	((Nutriti* adj (guideline* or panel* or table* or profil* or summar* or scor*)) or nutrition* fact* label*).mp.
5	(portion size* or serve or serving or serves).mp.
6	1 or 2 or 3 or 4
7	5 and 6
8	(animals not (humans and animals)).sh.
9	Autistic Disorder/ or autism.mp. or exp HIV/ or smoking.mp. or exp neoplasms/ or exp viruses/ or genetics/ or exp mental health/ or bioactive.mp. or osteoporosis/ or chronic pain/ or sexual.mp. or reproductive health/ or enzymes.mp. or malaria.mp. or bacterial infections/ or pathology/ or pathologic.mp. or vaccin*.mp. or immunization.mp. or zebrafish*.mp. or zebra fish.mp. or dengue.mp. or urinary.mp. or greenhouse.mp. or spores/ or kidney/ or kidney diseases/ or orthop?edic*.mp. or melanoma*.mp. or depression/ or tobacco.mp. or schizophrenia.mp. or pharmacy/ or radiology.mp. or rehabilitation/ or bacillus.mp. or chlamydia.mp. or thyroid gland/ or thyroid neoplasms/ or biobank.mp. or tooth diseases/ or genetic test*.mp. or botanical.mp. or infant mortal*.mp. or neonatal death*.mp. or neonatal mortalit*.mp. or infant death*.mp. or biometry/ or biometric identification/ or carcinoma.mp. or mercury/ or mercury.mp. or brain injur*.mp. or brain injuries/ or chloroplast*.mp. or autopsy.mp. or acquired immunodeficiency syndrome/ or pharmacological.mp. or biomedical.mp. or infectious disease*.mp. or botox.mp. or amino acid*.mp. or newborn screen*.mp. or pap test*.mp. or spectroscopy.mp. or glucometer*.mp. or yoghurt fermentation.mp. or staphylococcus.mp. or pharmacist*.mp. or domestic abuse*.mp. or catheterization.mp. or genetically modified.mp. or hypersensitivity/ or allergic reaction*.mp. or copper exposure.mp. or epilepsy.mp. or epileptic*.mp. or renal.mp. or toxicology.mp. or oral health.mp. or reproduction.mp. or chromatographic.mp. or hepatitis.mp. or fortification.mp. or endosomal.mp. or anti-inflammatory.mp. or malignant.mp. or scoliosis.mp. or transcriptome.mp. or testicular.mp. or oncology.mp. or ophthalmologist*.mp. or adhd.mp. or attention deficit disorder with hyperactivity/ or transgender.mp. or post traumatic stress*.mp. or posttraumatic stress*.mp. or infection control.mp. or pharmacometalb*.mp. or radiomics.mp. or hormonal.mp. or performance enhancing drug* performance enhancing substances/ or skin cancer*.mp. or cervical cancer*.mp. or foot ulcer*.mp. or neurocognitive.mp. or myocardial infarction.mp. or colorectal cancer*.mp. or bisexual.mp. or biomarker*.mp. or an?esthesia.mp. or ebola.mp. or anxiety.mp.
10	7 not (8 or 9)
**11**	**limit 10 to yr="2010 -Current"**

PsycINFO

#	Searches
1	("point of sale" or "point of purchase").mp.
2	((nutrition* or food* or "front of pack*" or health* or "back of pack*") adj2 (label* or rating* or symbol* or information* or logo*)).mp.
3	((menu* or food*) adj3 label*).mp.
4	((Nutriti* adj (guideline* or panel* or table* or profil* or summar* or scor*)) or nutrition* fact* label*).mp.
5	(portion size* or serve or serving or serves).mp.
6	1 or 2 or 3 or 4
7	5 and 6
8	(animals not (humans and animals)).sh.
9	Autistic Disorder/ or autism.mp. or exp HIV/ or smoking.mp. or exp neoplasms/ or exp viruses/ or genetics/ or exp mental health/ or bioactive.mp. or osteoporosis/ or chronic pain/ or sexual.mp. or reproductive health/ or enzymes.mp. or malaria.mp. or bacterial infections/ or pathology/ or pathologic.mp. or vaccin*.mp. or immunization.mp. or zebrafish*.mp. or zebra fish.mp. or dengue.mp. or urinary.mp. or greenhouse.mp. or spores/ or kidney/ or kidney diseases/ or orthop?edic*.mp. or melanoma*.mp. or depression/ or tobacco.mp. or schizophrenia.mp. or pharmacy/ or radiology.mp. or rehabilitation/ or bacillus.mp. or chlamydia.mp. or thyroid gland/ or thyroid neoplasms/ or biobank.mp. or tooth diseases/ or genetic test*.mp. or botanical.mp. or infant mortal*.mp. or neonatal death*.mp. or neonatal mortalit*.mp. or infant death*.mp. or biometry/ or biometric identification/ or carcinoma.mp. or mercury/ or mercury.mp. or brain injur*.mp. or brain injuries/ or chloroplast*.mp. or autopsy.mp. or acquired immunodeficiency syndrome/ or pharmacological.mp. or biomedical.mp. or infectious disease*.mp. or botox.mp. or amino acid*.mp. or newborn screen*.mp. or pap test*.mp. or spectroscopy.mp. or glucometer*.mp. or yoghurt fermentation.mp. or staphylococcus.mp. or pharmacist*.mp. or domestic abuse*.mp. or catheterization.mp. or genetically modified.mp. or hypersensitivity/ or allergic reaction*.mp. or copper exposure.mp. or epilepsy.mp. or epileptic*.mp. or renal.mp. or toxicology.mp. or oral health.mp. or reproduction.mp. or chromatographic.mp. or hepatitis.mp. or fortification.mp. or endosomal.mp. or anti-inflammatory.mp. or malignant.mp. or scoliosis.mp. or transcriptome.mp. or testicular.mp. or oncology.mp. or ophthalmologist*.mp. or adhd.mp. or attention deficit disorder with hyperactivity/ or transgender.mp. or post traumatic stress*.mp. or posttraumatic stress*.mp. or infection control.mp. or pharmacometalb*.mp. or radiomics.mp. or hormonal.mp. or performance enhancing drug* performance enhancing substances/ or skin cancer*.mp. or cervical cancer*.mp. or foot ulcer*.mp. or neurocognitive.mp. or myocardial infarction.mp. or colorectal cancer*.mp. or bisexual.mp. or biomarker*.mp. or an?esthesia.mp. or ebola.mp. or anxiety.mp.
10	7 not (8 or 9)
**11**	**limit 10 to yr="2010 -Current"**

CINAHL

Search Terms	Search options
**S6**	**S3 NOT S4 Published Date: 20100101-20190430**
S5	S3 NOT S4
S4	“Autistic Disorder” or autism or exp HIV or smoking or exp neoplasms or exp viruses or genetics or “mental health” or bioactive or osteoporosis or “chronic pain” or sexual or “reproductive health” or enzymes or malaria or “bacterial infections*” or pathology or pathologic or vaccin* or immunization or zebrafish* or “zebra fish” or dengue or urinary or greenhouse or spores or kidney or kidney diseases or orthop?edic* or melanoma* or depression or tobacco or schizophrenia or pharmacy or radiology ...
S3	S1 AND S2
S2	"portion size*" or serve or serving or serves
S1	“point of sale” or “point or purchase” or ((nutrition* or food* or "front of pack*" or health* or "back of pack*") adj2 (label* or rating* or symbol* or information* or logo*)) or ((menu* or food*) n3 label*) or ((Nutriti* n1 (guideline* or panel* or table* or profil* or summar* or scor*)) or “nutrition* fact* label*”)

Business Source Ultimate

Search Terms	Search Options
**S6**	**S3 NOT S4 Published Date: 20100101-20190430**
S5	S3 NOT S4
S4	“Autistic Disorder” or autism or exp HIV or smoking or exp neoplasms or exp viruses or genetics or “mental health” or bioactive or osteoporosis or “chronic pain” or sexual or “reproductive health” or enzymes or malaria or “bacterial infections*” or pathology or pathologic or vaccin* or immunization or zebrafish* or “zebra fish” or dengue or urinary or greenhouse or spores or kidney or kidney diseases or orthop?edic* or melanoma* or depression or tobacco or schizophrenia or pharmacy or radiology ...
S3	S1 AND S2
S2	"portion size*" or serve or serving or serves
S1	“point of sale” or “point or purchase” or ((nutrition* or food* or "front of pack*" or health* or "back of pack*") adj2 (label* or rating* or symbol* or information* or logo*)) or ((menu* or food*) n3 label*) or ((Nutriti* n1 (guideline* or panel* or table* or profil* or summar* or scor*)) or “nutrition* fact* label*”)

Cochrane

Search Name:
Description:

ID	Search
#1	("point of sale" or "point of purchase")
#2	((nutrition* or food* or "front of pack*" or health* or calorie* or energy) near/1 (label* or rating* or symbol* or information* or logo* or information))
#3	((menu* or food*) near/3 label*)
#4	(Nutriti* near/1 (value or fact* or information or guideline* or panel* or table* or profil* or summar* or scor*)) 1078
#5	[80-#4]
#6	"portion size*"
#7	serve
#8	serves
#9	serving
#10	[81-#9]
#11	[80, #10]
**#12**	**[80, #10] publication year from 2010 to 2019**

Scopus

(((TITLE-ABS-KEY ("point of purchase")) OR (TITLE-ABS-KEY (nutrition* W/2 rating*)) OR (TITLE-ABS-KEY (nutrition* W/2 symbol*)) OR (TITLE-ABS-KEY (nutrition* W/2 logo*))) OR ((TITLE-ABS-KEY ("Food labeling")) OR (TITLE-ABS-KEY ("Traffic light*")) OR (TITLE-ABS-KEY (label* W/5 star*)) OR (TITLE-ABS-KEY (food* W/2 label*)) OR (TITLE-ABS-KEY (health* W/2 information*)) OR (TITLE-ABS-KEY (food* W/2 information*)) OR (TITLE-ABS-KEY (label* W/5 percentage*))) OR ((TITLE-ABS-KEY ("front of pack*" W/2 label*)) OR (TITLE-ABS-KEY ("front of pack*" W/2 rating*)) OR (TITLE-ABS-KEY ("front of pack*" W/2 symbol*)) OR (TITLE-ABS-KEY ("front of pack*" W/2 information*)) OR (TITLE-ABS-KEY ("front of pack*" W/2 logo*)) OR (TITLE-ABS-KEY (nutrition* W/2 information*))) OR ((TITLE-ABS-KEY (nutrition* W/2 label*)) OR (TITLE-ABS-KEY (food* W/2 rating*)) OR (TITLE-ABS-KEY (food* W/2 symbol*)) OR (TITLE-ABS-KEY (food* W/2 logo*)) OR (TITLE-ABS-KEY ("point of sale")) OR (TITLE-ABS-KEY (health* W/2 rating*)) OR (TITLE-ABS-KEY (health* W/2 symbol*))) OR ((TITLE-ABS-KEY (health* W/2 logo*)) OR (TITLE-ABS-KEY ("back of pack*" W/2 label*)) OR (TITLE-ABS-KEY ("back of pack*" W/2 rating*)) OR (TITLE-ABS-KEY ("back of pack*" W/2 symbol*)) OR (TITLE-ABS-KEY ("back of pack*" W/2 information*)) OR (TITLE-ABS-KEY ("back of pack*" W/2 logo*)) OR (TITLE-ABS-KEY (menu* W/3 label*))) OR (TITLE-ABS-KEY (nutriti* W/5 scor*)) OR ((TITLE-ABS-KEY (health* W/2 label*)) OR (TITLE-ABS-KEY (nutrition* AND fact* AND label*)) OR (TITLE-ABS-KEY (nutriti* W/5 guideline*)) OR (TITLE-ABS-KEY (nutriti* W/5 panel*)) OR (TITLE-ABS-KEY (nutriti* W/5 table*)) OR (TITLE-ABS-KEY (nutriti* W/5 summar*)) OR (TITLE-ABS-KEY (nutriti* W/5 profil*))))) AND ("portion size*" OR TITLE-ABS-KEY (serves OR serving OR serve)) AND NOT ((autistic AND disorder) OR (autism OR hiv OR smoking OR neoplasms OR viruses OR genetics) OR (bioactive OR osteoporosis OR sexual OR enzymes OR malaria OR pathology OR pathologic OR vaccin* OR immunization OR dengue OR urinary OR greenhouse OR spores OR kidney OR melanoma* OR depression OR tobacco OR schizophrenia OR pharmacy OR radiology OR rehabilitation OR bacillus OR chlamydia OR biometry OR biobank OR chloroplast* OR autopsy OR zebrafish* OR spectroscopy OR glucometer* OR staphylococcus OR pharmacist* OR catheterization OR carcinoma OR mercury OR pharmacological OR biomedical OR botox OR epilepsy OR epileptic* OR renal OR toxicology OR hypersensitivity OR reproduction OR chromatographic OR hepatitis OR fortification OR endosomal OR anti-inflammatory OR malignant OR scoliosis OR transcriptome OR testicular OR oncology OR ophthalmologist* OR adhd OR transgender OR pharmacometalb* OR radiomics OR hormonal OR bisexual OR biomarker* OR an?esthesia OR ebola OR anxiety) OR ("mental health" OR "chronic pain" OR "reproductive health" OR "bacterial infections") OR ("zebra fish" OR "kidney diseases" OR "thyroid gland" OR "thyroid neoplasms" OR "tooth diseases" OR "genetic test*" OR "infant mortal*" OR "neonatal death*" OR "neonatal mortalit*" OR "infant death*") OR ("biometric identification" OR "brain injur*" OR "brain injuries" OR "acquired immunodeficiency syndrome" OR "infectious disease*" OR "amino acid*" OR "newborn screen*" OR "pap test*" OR "yoghurt fermentation" OR "domestic abuse*" OR "genetically modified" OR "allergic reaction*" OR "copper exposure" OR "oral health" OR "attention deficit disorder with hyperactivity" OR "post traumatic stress*" OR "posttraumatic stress*" OR ' "infection control" OR "performance enhancing drug*" OR "performance enhancing substances" OR "skin cancer*" OR "cervical cancer*" OR "foot ulcer*" OR "myocardial infarction" OR "colorectal cancer*") OR (orthop?edic* OR botanical OR neurocognitive)) AND (LIMIT-TO (PUBYEAR , 2019) OR (LIMIT-TO (PUBYEAR , 2018) OR (LIMIT-TO (PUBYEAR , 2017) OR LIMIT-TO (PUBYEAR , 2016) OR LIMIT-TO (PUBYEAR , 2015) OR LIMIT-TO (PUBYEAR , 2014) OR LIMIT-TO (PUBYEAR , 2013) OR LIMIT-TO (PUBYEAR , 2012) OR LIMIT-TO (PUBYEAR , 2011) OR LIMIT-TO (PUBYEAR , 2010))
